# In Vivo Renin Activity Imaging in the Kidney of Progeroid *Ercc1* Mutant Mice

**DOI:** 10.3390/ijms222212433

**Published:** 2021-11-18

**Authors:** Bibi S. van Thiel, Janette van der Linden, Yanto Ridwan, Ingrid M. Garrelds, Marcel Vermeij, Marian C. Clahsen-van Groningen, Fatimunnisa Qadri, Natalia Alenina, Michael Bader, Anton J. M. Roks, A. H. Jan Danser, Jeroen Essers, Ingrid van der Pluijm

**Affiliations:** 1 Department of Molecular Genetics, Cancer Genomics Center, Erasmus University Medical Center, 3015GD Rotterdam, The Netherlands; bibivanthiel@gmail.com (B.S.v.T.); j.vanderlinden@erasmusmc.nl (J.v.d.L.); r.ridwan@erasmusmc.nl (Y.R.); 2 Division of Vascular Medicine and Pharmacology, Department of Internal Medicine, Erasmus University Medical Center, 3015GD Rotterdam, The Netherlands; i.vandenberg-garrelds@erasmusmc.nl (I.M.G.); a.roks@erasmusmc.nl (A.J.M.R.); a.danser@erasmusmc.nl (A.H.J.D.); 3 Department of Vascular Surgery, Erasmus University Medical Center, 3015GD Rotterdam, The Netherlands; 4 Department of Experimental Cardiology, Erasmus University Medical Center, 3015GD Rotterdam, The Netherlands; 5 Department of Pathology, Erasmus University Medical Center, 3015GD Rotterdam, The Netherlands; vermeijmarcel@gmail.com (M.V.); m.clahsen-vangroningen@erasmusmc.nl (M.C.C.-v.G.); 6 Max Delbrück Center, 13125 Berlin, Germany; fqadri@mdc-berlin.de (F.Q.); alenina@mdc-berlin.de (N.A.); mbader@mdc-berlin.de (M.B.); 7 DZHK (German Center for Cardiovascular Research), Partner Site Berlin, 10785 Berlin, Germany; 8 Charité—University Medicine, 10117 Berlin, Germany; 9 Institute for Biology, University of Lübeck, 23562 Lübeck, Germany; 10 Department of Radiation Oncology, Erasmus University Medical Center, 3015GD Rotterdam, The Netherlands

**Keywords:** renin-angiotensin system, in vivo imaging, renin, renal disease, renal aging

## Abstract

Changes in the renin–angiotensin system, known for its critical role in the regulation of blood pressure and sodium homeostasis, may contribute to aging and age-related diseases. While the renin–angiotensin system is suppressed during aging, little is known about its regulation and activity within tissues. However, this knowledge is required to successively treat or prevent renal disease in the elderly. Ercc1 is involved in important DNA repair pathways, and when mutated causes accelerated aging phenotypes in humans and mice. In this study, we hypothesized that unrepaired DNA damage contributes to accelerated kidney failure. We tested the use of the renin-activatable near-infrared fluorescent probe ReninSense680™ in progeroid *Ercc1*^*d*/−^ mice and compared renin activity levels in vivo to wild-type mice. First, we validated the specificity of the probe by detecting increased intrarenal activity after losartan treatment and the virtual absence of fluorescence in renin knock-out mice. Second, age-related kidney pathology, tubular anisokaryosis, glomerulosclerosis and increased apoptosis were confirmed in the kidneys of 24-week-old *Ercc1*^*d*/−^ mice, while initial renal development was normal. Next, we examined the in vivo renin activity in these *Ercc1*^*d*/−^ mice. Interestingly, increased intrarenal renin activity was detected by ReninSense in *Ercc1*^*d*/−^ compared to WT mice, while their plasma renin concentrations were lower. Hence, this study demonstrates that intrarenal RAS activity does not necessarily run in parallel with circulating renin in the aging mouse. In addition, our study supports the use of this probe for longitudinal imaging of altered RAS signaling in aging.

## 1. Introduction

Aging is a natural biological process that is associated with diverse detrimental changes in cells and tissues, ultimately leading to loss of organ function. Progressive deterioration of the renal structure is part of the normal aging process, including loss of renal mass, loss of tubules and increases in the amounts of glomerulosclerosis and tubulointerstitial fibrosis [1]. In addition to sclerosis and subsequent loss of many of the glomeruli, the remaining glomeruli often exhibit impaired filtration ability. Accordingly, many elderly show a decline in renal function, often shown as progressive decreases in glomerular filtration rate and renal blood flow. These age-related structural and functional changes may predispose the kidneys to acute kidney injury or progressive chronic kidney disease [2].

The renin–angiotensin system (RAS) has long been recognized for its critical role in the regulation of blood pressure and fluid homeostasis. Changes in the responsiveness and activity of the RAS have been shown to play an important role in aging, as well as in renal disease, as they predispose the elderly to acute kidney injury and chronic kidney disease [3,4,5,6,7]. It is suggested that overexposure to the RAS hormone angiotensin (Ang) II causes DNA damage, as well as cellular senescence or apoptosis, processes known to play a role in aging and disease [8,9]. Moreover, interference in the RAS system by using RAS blockers has been proposed to extend the lifespan and prevent age-associated changes [10]. However, not all elderly respond well to RAS blockade and related adverse events include acute kidney injury, hyperkalemia and hypotension [11,12]. Thus, we need more insight into the regulation of the RAS during aging in order to successively treat or prevent renal disease in the elderly population.

Although Ang II is considered to be the principal effector molecule of the RAS, renin is the rate-limiting enzyme in the cascade, which plays an essential role in regulating RAS activity. Several drugs blocking renin activity have been shown to have renoprotective actions [13]. Currently, plasma renin activity is used as the clinical marker for systemic RAS activity, and previous studies have shown that circulating renin is suppressed with advancing age [7,14]. However, multiple studies have reported on the existence of so-called tissue RAS, which may act independently of the systemic RAS [15]. Indeed, RAS components in the kidneys do not always change in parallel with RAS components in circulation [16]. In fact, inappropriate activation of the intrarenal RAS might underlie the pathogenesis of hypertension and renal injury (reviewed by Kobori et al.) [17]. Thus, next to systematic plasma renin activity measurements, more emphasis should be placed on quantifying tissue RAS activity. As it is difficult to measure tissue RAS components in vivo, non-invasive imaging of local renin activity would help to evaluate the possible role of tissue renin activity in disease development and progression. Moreover, the development of new non-invasive imaging methods with the use of near-infrared fluorescent (NIRF) probes could lead to better detection and treatment options in the future.

In this study, we used a DNA-repair-compromised mutant mouse model based on the nucleotide excision repair gene Ercc1 [18]. Together with Xpf, Ercc1 forms an endonuclease required to resolve DNA intra- and interstrand cross-links, and a subset of homologous recombination intermediates [19] and mutations in this gene are, thus, compromised in multiple DNA repair pathways. The combined genotype of the null and the seven-amino-acid deletion of the Ercc1 gene (*Ercc1*^*d*/−^) in this mouse model has been shown to develop a broad spectrum of aging-related changes that are also observed in wild-type mice, but with varying rates between lesions [20]. It has previously been shown that the kidneys of the progeroid *Ercc1*^*d*/−^ mouse model display severe tubular attenuation and degeneration with marked anisokaryosis [20,21]. Moreover, Schermer et al. [22] showed that age-related transcriptional changes were present in the glomeruli of *Ercc1*^*d*/−^ mice, suggesting that the progeroid *Ercc1*^*d*/−^ mouse model is a valuable tool for studying age-related glomerular pathologies. The NIRF probe ReninSense680™ contains an angiotensinogen-derived peptide sequence flanked by two NIR fluorochromes that produces a fluorescent signal after cleavage by renin produced in the kidneys. This probe may be used to monitor abnormal RAS function, progression of disease and the efficacy of therapeutic treatment in hypertension and cardiovascular disease [23]. To investigate age-related changes in the intrarenal RAS in vivo, we applied the renin-activatable NIRF probe ReninSense680™, allowing non-invasive imaging of renin activity in the progeroid *Ercc1*^*d*/−^ mouse model [23].

## 2. Results

### 2.1. Progeroid Ercc1^d/−^ Mice Display Age-Related Kidney Pathology

**Figure 1:**
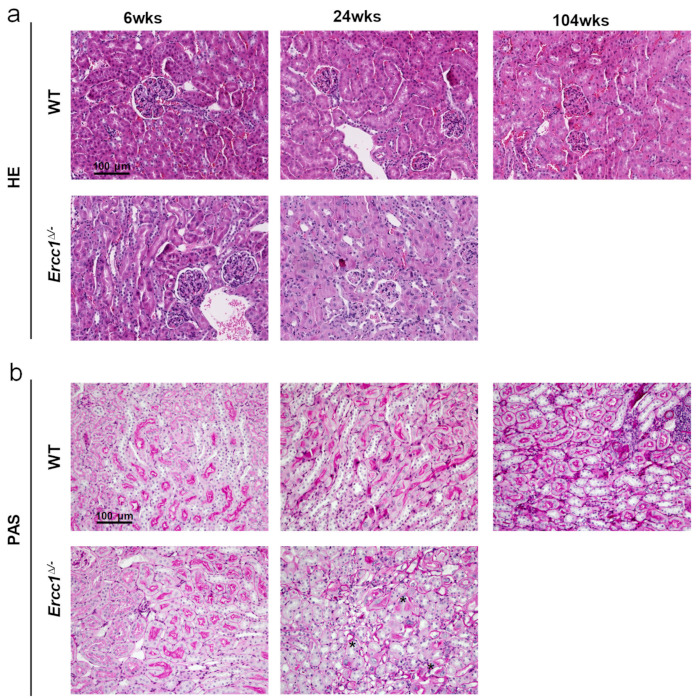
Histopathological changes in the kidneys of progeroid *Ercc1*^*d*/−^ mice. Representative pictures of hematoxylin and eosin (HE) (**a**), and periodic acid–Schiff (PAS) staining (**b**) of the kidneys of 6- and 24-week-old *Ercc1*^*d*/−^ mice, their wild-type (WT) littermates and old WT mice (104 weeks of age). Histological examination showed signs of kidney aging, including anisokaryosis, tubular degeneration, loss of brush borders and glomerulosclerosis in kidneys of *Ercc1*^*d*/−^ mice at 24 weeks of age (indicated by * in the PAS staining). In all panels, scale bar = 100 μm.

**Figure 2:**
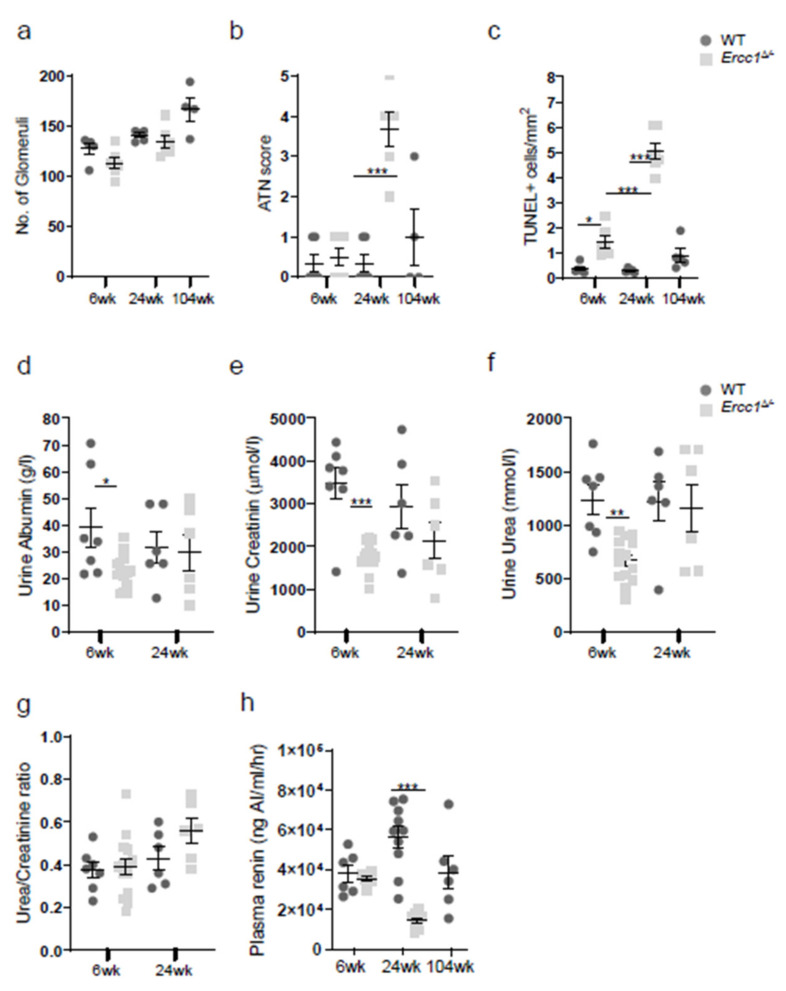
Functional renal changes in progeroid *Ercc1*^*d*/−^ mice. (**a**) The number of glomeruli confirmed normal kidney development from birth in *Ercc1*^*d*/−^. (**b**) ATN scoring showed significantly increased kidney damage in 24-week-old *Ercc1*^*d*/−^ mice compared to their wild-type (WT) littermates. (**c**). Quantification of TUNEL staining indicated significantly increased apoptotic cell death in *Ercc1*^*d*/−^ kidneys. Urinary albumin (**d**), creatinine (**e**) and urea (**f**) levels were comparable between 24-week-old *Ercc1*^*d*/−^ and WT mice. (**g**) Urea/creatinine ratio showed no significant differences between *Ercc1*^*d*/−^ and WT mice. (**h**) Plasma renin concentration was significantly decreased in 24-week-old *Ercc1*^*d*/−^ mice compared to WT. Data are means ± SEM of n = 4–6 (**a**–**c**) and n = 6–14 (**d**–**h**). Note: * *p* < 0.05, ** *p* < 0.01, *** *p* < 0.001 vs. WT.

We first set out to confirm the age-related kidney pathology in *Ercc1*^*d*/−^ mice, for which we examined kidneys of 6, 24 and 104 (WT only)-week-old mice. Renal development of *Ercc1*^*d*/−^ kidneys was found to be normal, as at 6 weeks of age *Ercc1*^*d*/−^ animals displayed normal kidney architecture, including normal numbers of glomeruli (Figure 1a and Figure 2a). However, *Ercc1*^*d*/−^ mice display progressive kidney pathology, including tubular degeneration, loss of brush borders and anisokaryosis (Figure 1), and a significantly increased acute tubular necrosis (ATN) score (Figure 2b). In addition, they present with signs of kidney aging, as shown by reduced proliferation (data not shown) and increased apoptosis (Figure 2c) at 24 weeks of age. We measured urinary albumin, creatinine and urea levels, which were comparable in *Ercc1*^*d*/−^ mice compared to WT mice at 24 weeks of age (Figure 2d–g). This ruled out significant renal dysfunction. At 6 weeks of age, urinary albumin, creatinine and urea levels were significantly lower in *Ercc1*^*d*/−^ mice compared to WT littermates, although urea/creatinine ratio levels were comparable to WT mice, which did not imply renal dysfunction. Remarkably, while normal plasma renin concentrations were found at 6 weeks of age, plasma renin concentrations in the *Ercc1*^*d*/−^ mice were significantly lower compared to WT mice at 24 weeks of age (Figure 2h).

### 2.2. ReninSense Selectively Detects Renin Activity in the Kidney In Vitro

**Figure 3:**
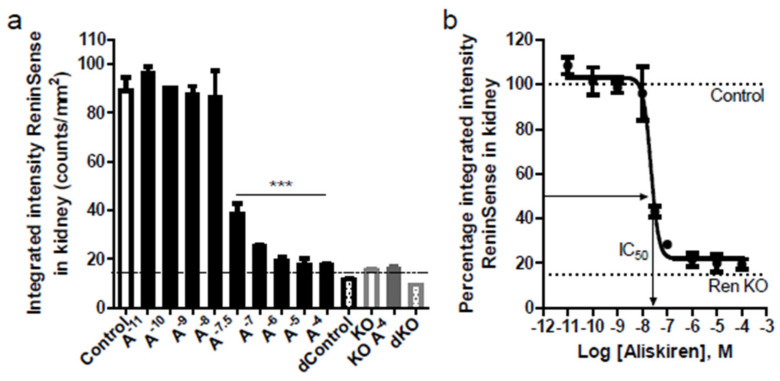
Specific in vitro enzymatic activation of ReninSense by kidney and plasma renin. (**a**) ReninSense was rapidly activated by kidney renin in WT mice. Low levels of fluorescence were found in RenKO kidney lysates comparable to the autofluorescence of the probe. High concentrations of aliskiren completely blocked ReninSense activation. (**b**) The half-maximal inhibitory concentration (IC50) for aliskiren in kidney lysates was 10^−7.7^ M. Data are means ± SEM of duplicate samples. Note: *** *p* < 0.001 vs. control.

To assess the ability of ReninSense680™ to detect both kidney and plasma renin, the activation of ReninSense was tested in kidney lysates and plasma from WT and Ren1c homozygous null (RenKO) mice, with and without co-incubation of the renin inhibitor aliskiren. As expected, ReninSense was rapidly activated in kidney lysates of WT mice as assessed by fluorescent measurements with the odyssey system. The microplate kidney extract fluorescent assay showed <5% variation between duplicate wells. Aliskiren blocked ReninSense activation in a concentration-dependent manner by ≈80% (Figure 3a). The half-maximal inhibitory concentration (IC50) for aliskiren in kidney lysates was approximately 10^−7.7^ M, as measured here with the ReninSense probe (Figure 3b), i.e., close to the IC50 reported earlier for mouse renin [24]. The remaining fluorescent signal in the presence of the highest concentration of aliskiren was comparable to the fluorescence seen in kidney extracts from RenKO mice and denatured kidneys, indicating that this is the background fluorescent level of the ReninSense probe—in other words, the detection limit of this system. When evaluating the ReninSense probe in mouse plasma, fluorescence levels remained in this background range and were unaffected by aliskiren, indicating that the probe cannot be used to measure renin activity in plasma using the odyssey system.

### 2.3. In Vivo Imaging of Renin Upregulation Shown by ReninSense

**Figure 4:**
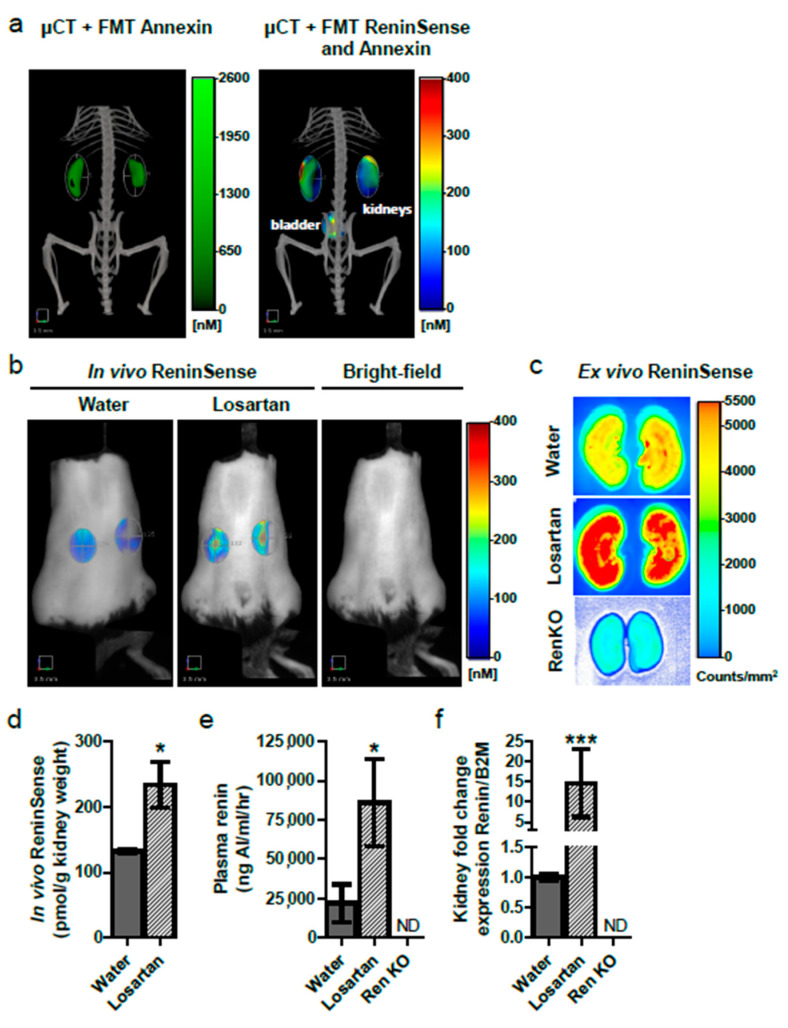
In vivo activation of ReninSense in kidneys of WT mice, with and without losartan treatment. (**a**) Mice were imaged tomographically by FMT 2500 and microCT 24 h after ReninSense injection. Micro CT imaging and FMT imaging of Annexin-Vivo allowed accurate localization of the kidneys. Combined microCT and FMT imaging of Annexin-Vivo and ReninSense showed in vivo renin activity in the kidneys and bladder (clearance of probe). (**b**) Losartan-treated mice showed increased in vivo intrarenal renin activity, which was confirmed by quantification. (**c**,**d**) Ex vivo imaging of the kidneys by the Odyssey^®^ system confirmed activation of the ReninSense probe in losartan-treated mice. Fluorescence of ReninSense could not be detected in vivo or ex vivo in RenKO mice. (**e**) Losartan treatment increased plasma renin activity. (**f**) Increased expression levels of renin in the kidneys were found in losartan-treated mice. ND, not detectable. Data are means ± SEM of n = 3. Note: * *p* < 0.05, *** *p* < 0.01 vs. WT.

To address the ability of ReninSense to be cleaved and used as a readout for in vivo renin activity, ReninSense activation was examined in WT mice treated either with vehicle or with the Angiotensin II receptor type 1 (AT_1_ receptor) antagonist losartan, which is known to increase renin levels. In addition, ReninSense activation was measured in Ren1c homozygous null (RenKO) mice. Animals were imaged tomographically by FMT 2500 24 h after ReninSense injection. To improve the detection of intrarenal renin activity, mice were injected with the NIRF probe Annexin-Vivo750™ to visualize the kidneys, and when possible were also imaged with the microCT to allow co-registration of anatomical data with the in vivo fluorescence (Figure 4a). Losartan-treated mice showed increased in vivo (Figure 4b) and ex vivo (Figure 4c) activation of ReninSense in their kidneys compared to vehicle treated mice. The increase in renin activity after losartan treatment was validated by quantification of the in vivo results (Figure 4d), increased plasma renin activity (Figure 4e) and increased renin expression levels in the kidneys (Figure 4f). As expected, fluorescence of ReninSense could not be detected in vivo or ex vivo in RenKO mice, which do not express the renin gene. These results validate the specificity of the ReninSense probe for renin activity.

### 2.4. Increased Renin Activity in the Kidneys of Progeroid Ercc1^d/−^ Mice In Vivo

**Figure 5:**
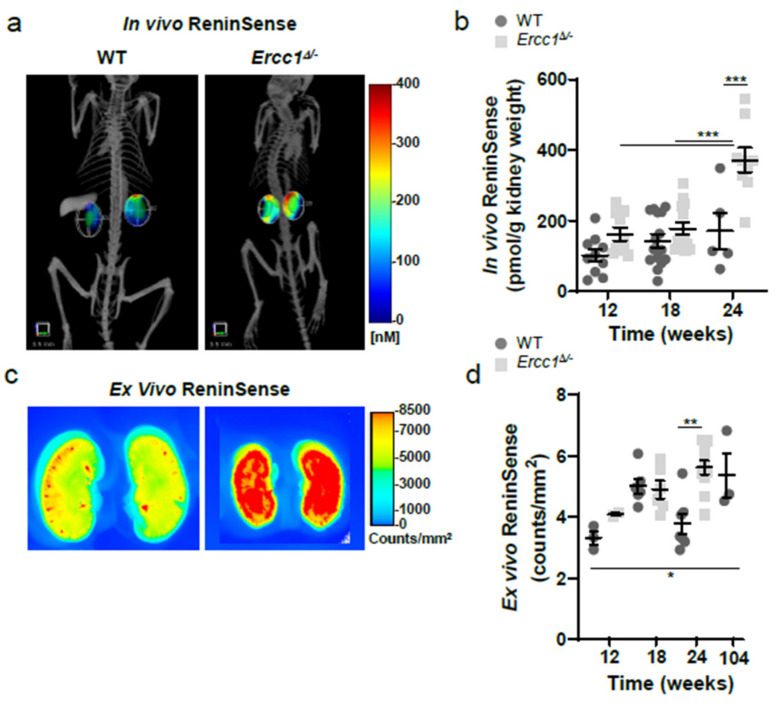
In vivo imaging of renin activity by ReninSense in progeroid *Ercc1*^*d*/−^ mice. (**a**) *Ercc1*^*d*/−^ mice display activated intrarenal renin activity at 24 weeks of age, as evidenced by increased fluorescence detected with the ReninSense probe when imaged with the microCT and FMT. (**b**) Quantification of the in vivo fluorescence of ReninSense confirmed increased renin activity in the kidneys, which was significantly different at 24 weeks of age. (**c**) These results were further confirmed ex vivo by imaging of the 24-week-old kidneys with the Odyssey^®^ system. Data are means ± SEM of n = 5–15 (**b**) and n = 3–12 (**d**). Differences were assessed by one-way ANOVA, followed by correction for multiple testing by post hoc Bonferroni analysis. Note: * *p* < 0.05, ** *p* < 0.01, *** *p* < 0.001 vs. WT.

While it is generally accepted that circulating renin is suppressed during aging, little is known about the regulation or activity of renin within tissues with increasing age. In order to investigate in vivo kidney renin activity during aging, we injected progeroid *Ercc1*^*d*/−^ mice and their WT littermates with ReninSense at 12, 18 and 24 weeks of age. Combined microCT and FMT imaging of ReninSense showed increased in vivo intrarenal renin activity in *Ercc1*^*d*/−^ mice compared to WT mice from 12 weeks of age onwards, which was significantly different at 24 weeks of age (Figure 5a,b). Quantification of the in vivo fluorescence (Figure 5b) and ex vivo imaging of the kidneys (Figure 5c,d) confirmed these results. We found no differences in in vivo renin activity between male and female mice.

## 3. Discussion

Changes in the RAS are associated with the pathophysiology of various cardiovascular and renal diseases; therefore, targeting the RAS seems a logical therapeutic approach in the treatment of these diseases. Indeed, pharmacological RAS blockade has been shown to effectively slow down the progression of renal disease. However, it is important to note that not all patients (e.g., the elderly) respond well to RAS blockade. While the systemic RAS is suppressed with advancing age, the regulation and activity of tissue RAS during aging is not well defined. As such, previous reports have showed that although the circulating RAS is suppressed during normal aging, some components of the intrarenal RAS are elevated [3,16,17,25,26,27]. Varying tissue RAS activity might, at least in part, explain why elderly respond unpredictable to RAS blockade. Therefore, in this study we aimed to evaluate the use of the renin-activatable near-infrared fluorescent probe ReninSense to facilitate non-invasive imaging of renin activity in vivo. In addition, we investigated the activity levels of plasma and intrarenal renin in progeroid *Ercc1*^*d*/−^ mice with accompanying age-related kidney pathology. First, we showed that ReninSense specifically detects renin activity, as the fluorescence of the probe was increased after losartan treatment, while virtually no fluorescence could be detected in RenKO mice. Secondly, this study demonstrated that intrarenal renin activity does not necessarily run in parallel with circulating renin in the progeroid aging *Ercc1*^*d*/−^ mice.

It is important to note that most of the clinical studies supporting the beneficial effects of RAS inhibition do not include participants older than 75 years of age or elderly patients that are frail and with a high comorbidity burden [28,29]. Not all elderly respond well to RAS blockade, and related adverse events include acute kidney injury, hyperkalemia, hypotension and a further decline in glomerular filtration rate [3,11,12,30,31,32]. Additionally, combination therapy with ACE inhibitors and AT_1_ receptor blockers in patients with cardiovascular complications is linked to an increased risk of adverse renal outcomes with higher rates of hyperkalemia, hypotension and renal dysfunction and no observed benefit with respect to overall mortality [33,34,35,36]. The occurrence of these side effects might be worse in the elderly population, as they are prone to developing acute kidney injury and hyperkalemia due to the risk of complete RAS inhibition, as they already have low plasma renin levels. Therefore, caution and close monitoring are recommended when using these drugs in elderly patients with kidney dysfunction, and the optimal RAS inhibition with respect to end organ protection has yet to be determined in the elderly [37]. In this respect, it would be interesting to see how RAS inhibition would affect the aging kidneys alone; in other words, to study the effects of RAS inhibition in kidney-specific *Ercc1* mutant mice, which would represent a healthy mouse with aging kidneys. This might answer important questions on how the RAS is regulated in the aging kidneys and whether the effects of RAS blockers are systemic or not.

Controversy remains as to whether all RAS components that are required to generate Ang II are produced locally or are taken up from the circulation [15,38,39]. In the present study, the opposing findings on intrarenal and plasma renin in progeroid *Ercc1*^*d*/−^ mice supports an independent upregulation of intrarenal RAS. Additionally, urine albumin, creatinine and urea levels in these mice did not indicate renal dysfunction, yet the histopathological analysis clearly showed increased kidney damage. This might be very similar in the elderly, as their circulating renin decreases with increasing age, which could imply that renal renin activity is a very early aging marker [7,14]. It remains to be seen whether kidney renin levels increase with age in the elderly population. The fluorescence of the ReninSense near-infrared probe can be detected in deep tissue to a maximum penetration depth of 1–2 cm due to light attenuation caused by light absorption and scattering. Moreover, the currently used probe is a cleavable substrate, showing fluorescence upon cleavage by renin. In the clinic, radioactive PET/SPECT radiotracer molecules are mostly used, which are incorporated or accumulate at the site of interest; cleavable substrates are not yet used. Hence, in its current form, the probe would not be applicable to clinical investigations. Interestingly, low plasma renin levels with increased kidney renin levels have also been found in diabetic patients [13,40]. Animal models of early diabetic nephropathy identically showed decreased plasma renin activity and increases in kidney renin [41,42,43,44]. Epidemiologic studies showed that with age, the incidence and susceptibility of abnormal glucose levels and diabetic disease increase, although the mechanisms linking aging and diabetes are not well understood [45,46]. It is suggested that increased intrarenal renin is responsible for the development and progression of nephropathy in diabetes through increased intrarenal AT_1_ receptor signaling [17,41]. Therefore, it would be interesting to investigate whether diabetes is responsible for this increased intrarenal renin and accompanying kidney injury, or rather that this increased intrarenal RAS, as with diabetes, is in fact an concomitant result of the aging process [40].

As the circulating RAS does not necessarily reveal the responsiveness of the RAS within tissues, there is a need for reliable methods to assess the RAS within tissues. Whether urinary angiotensinogen reflects intrarenal RAS activity is doubtful [47,48,49]; recent data suggest that urinary angiotensinogen is plasma-derived, and that its variation in urine is determined by megalin-mediated reabsorption [50,51]. In addition, renal plasma flow responses to infused Ang II are used as an indirect measure of intrarenal RAS activation in humans, as this correlates inversely with endogenous RAS activity [52,53,54,55,56]. However, all of these methods are indirect measurements of intrarenal RAS activity, and currently there is no method to directly assess intrarenal RAS activity in humans. Thus, non-invasive imaging of the ReninSense probe holds considerable promise for improving the detection and localization of local renin activity, including intrarenal renin. Determining local renin activity would help to evaluate the complexity of RAS biology and the possible role of local renin activity in disease development and progression. Moreover, this method enables longitudinal imaging of altered RAS signaling; consequently, disease progression can be monitored over time and the effects of (new) interventions can be studied non-invasively.

In the present study, the fluorescence levels of the ReninSense probe in mouse plasma remained in the background range and were unaffected by aliskiren, indicating that the probe cannot be used to measure plasma renin activity with the odyssey system. These results are consistent with the results demonstrated by Zhang et al., as ReninSense fluorescence in mouse plasma in their study was also unaffected by renin inhibition [23]. Only when mice were treated with a low-salt diet did ReninSense fluorescence increase over time, while renin inhibitor treatment in these mice reduced the fluorescence to a level similar to the fluorescence levels in untreated mouse plasma, indicating that the measured fluorescence in normal mouse plasma actually represented the background level. We did, however, observe that ReninSense was rapidly activated in kidney lysates of WT mice and that aliskiren blocked ReninSense activation by ≈80%. The remaining fluorescent signal in the presence of the highest concentration of aliskiren was comparable to the fluorescence seen in kidney extracts from RenKO mice and denatured kidneys. This implies that the remaining fluorescent signal either represents the background fluorescent level of the ReninSense probe or represents activation of the probe ReninSense by renin-like enzymes (e.g., cathepsins), which might also be capable of reacting with the angiotensinogen sequence of the probe. Nevertheless, when comparing in vivo and ex vivo kidney activation of ReninSense in RenKO mice, fluorescence did not reach the threshold value and could not be detected, while losartan significantly increased kidney fluorescence levels in vivo as well as ex vivo, verifying the specificity of the probe to measure renin activity in the kidneys of small animals. Interestingly, the fluorescence signal is observed uniformly throughout the entire kidney, i.e., in both cortex and medulla. This coincides with the observation that Ang II levels in cortex and medulla are similar [57] and supports the concept of renin acting in the interstitial space, where it occurs at high levels after being released from cortical juxtaglomerular cells. Based on these data, it is unlikely that the probe detects stored renin, since its fluorescent signal in the cortex should be far above that in the medulla. This is reassuring, since stored renin does not yield angiotensins—this requires its release, allowing contact with angiotensinogen. This would imply that the probe does not accumulate in cells and acts extracellularly only, i.e., at the relevant site of angiotensin generation [58].

In conclusion, we have demonstrated that the NIRF probe ReninSense can be used to non-invasively visualize and measure intrarenal renin activity. By using this method to identify local RAS activity, we might gain important insights into the changes in the RAS that occur with age, as well as in other (age-related) diseases. Although further study is warranted, our observations in the progeroid *Ercc1*^*d*/−^ mouse model provide evidence that circulating RAS activity does not necessarily run in parallel with intrarenal RAS activity during aging, which has important clinical consequences. Since increased intrarenal RAS activity might contribute to the disturbed kidney pathology observed in these mice, future investigations should examine the effects of the observed age-dependent changes in intrarenal renin activity on kidney deterioration.

## 4. Materials and Methods

All animal experiments were performed under the regulation and permission of the Animal Care Committee, conforming to the Guide for the Care and Use of Laboratory Animals published by the US National Institutes of Health (NIH Publication No. 8523, revised 1985). As required by Dutch law, formal permission to generate and use genetically modified animals was obtained from the responsible local and national authorities (DEC 118-11-05 and DEC 139-12-16).

### 4.1. Experimental Animals

Animals used in this study were male and female *Ercc1*^*d*/−^ mutants and their wild-type *Ercc1*^+/+^ littermates (WT) in an F1 hybrid FVB/N-C57BL/6J background. The generation of nucleotide excision repair-deficient *Ercc1*^*d*/−^ mice has been previously described [18]. Ren1c homozygous null mice (RenKO; 3 females and 1 male) were generated as described before (C57BL/6J background) and sacrificed at the age of 3–6 months [59]. A separate group of WT mice was divided into two groups, which were either given losartan (100 mg/kg/day) in drinking water or drinking water only from 5 weeks of age until the age of 12 weeks, when the animals were sacrificed.

All mice were housed under standard laboratory conditions (temperature 23 ± 1 °C, 12-h light-dark cycle) and maintained on standard chow (Special Diets Services, Essex, UK) with ad libitum access to water. Since *Ercc1*^*d*/−^ mice are smaller, water bottles with long nozzles were used and food was administered within the cages from four weeks of age. For each experiment with mutant animals, littermate controls were used unless stated otherwise. Animals were housed at the Animal Resource Centre (Erasmus University Medical Centre), which operates in compliance with the “Animal Welfare Act” of the Dutch government, using the “Guide for the Care and Use of Laboratory Animals” as its standard. As required by Dutch law, formal permission to generate and use genetically modified animals was obtained from the responsible local and national authorities. An independent Animal Ethics Committee consulted by Erasmus Medical Center (Stichting DEC Consult) approved these studies (permit number EMC2413), in accordance with national and international guidelines. For the described experiments, animals were sacrificed by CO_2_ inhalation, unless stated otherwise.

### 4.2. Histological Assessment

Emersion-formalin-fixated kidneys were embedded in paraffin, sectioned at 5 μm and mounted on Superfrost Plus slides. Cross-sections of the whole kidney, including the cortex and medulla, were stained according to standard diagnostic protocol for hematoxylin and eosin (HE) and periodic acid–Schiff stain (PAS) staining. In addition, TUNEL (terminal deoxynucleotidyl transferase-mediated dUTP nick end labelling; ApopTag In Situ Apoptosis Kit (Sigma-Aldrich S7100, Amsterdam, The Netherlands)) was performed on a 5μm section according to the manufacturer’s protocol. Acute kidney damage, as determined by tubular cell necrosis in the corticomedullary junction (scores ranged from mild (0) to extensive (5) damage), was assessed by a renal pathologist in a blinded fashion. Interstitial fibrosis in the cortical area was scored as 0 (<5% fibrosis), 1 (6–25% fibrosis), 2 (26–50% fibrosis) or 3 (>50% fibrosis) [60,61]. The number of TUNEL-positive cells in the kidneys was determined using 40× magnification.

### 4.3. Plasma Renin Concentration Measured by Enzyme-Kinetic Assay

To determine the plasma renin concentration, Ang I generation was quantified in the presence of excess sheep angiotensinogen [62]. Importantly, this approach does not detect prorenin, as it relies fully on enzymatic activity, while prorenin is enzymatically inactive.

### 4.4. Urine Measurements Relevant to Renal Function

Urine was collected and urinary protein, creatinine and urea levels were measured according to supplier instructions with a Pierce BCA Protein Assay Kit (Thermo Fisher Scientific, Rockford, IL, USA), QuantiChrome Creatinine Assay Kit (Gentaur, Brussels, Belgium) and QuantiChrome Urea Assay Kit (Gentaur, Brussels, Belgium), respectively.

### 4.5. In Vivo MicroCT-FMT Imaging of Renin Activity

*Ercc1*^*d*/−^ and WT mice, treated with or losartan or placebo, were injected intravenously with ReninSense680™ (2 nmol/100 μL per 25 g bodyweight) (Perkin Elmer Inc., Waltham, MA, USA) 24 h prior to FMT imaging. Mice were anesthetized (1.5–2.5% isoflurane, O_2_ 1 L/min) and depilated to minimize the interference of fur on the fluorescent signal. To improve detection of intrarenal renin activity, mice were injected with the NIRF probe Annexin-Vivo750™ (Perkin Elmer Inc., Waltham, MA, USA) 2 h prior to FMT imaging to visualize the kidneys or were imaged with the microCT to allow co-registration of anatomical data with the in vivo fluorescence. Before FMT imaging, mice were injected in the tail vein with the iodine contrast agent eXIA160 (Binitio Biomedical Inc., Ottawa, ON, Canada) for microCT imaging. Mice were positioned in the animal imaging cassette, restrained to prevent movement during imaging and imaged by using the Quantum FX imaging system (microCT) (Perkin Elmer Inc., Waltham, MA, USA). After microCT imaging, mice remained under anesthesia and the cassette was transferred to the FMT 2500 fluorescence tomography in vivo imaging system (Perkin Elmer Inc., Waltham, MA, USA). FMT imaging was performed using 680 and 750 nm excitation and emission wavelengths, respectively, 24 h after injection of ReninSense680™. The optimal re-injection time is 4 days, allowing for complete clearance of the agent from the mouse. Hence, the probe can be used for longitudinal imaging and is non-toxic. The multimodal animal imaging cassette facilitates the co-registration of microCT and FMT data through fiducial landmarks. Fusion of microCT and FMT images was done using the TrueQuant 4.0 software (Perkin Elmer Inc., Waltham, MA, USA). The position of the kidneys was determined via the fluorescence of Annexin-Vivo 750™ in the kidneys or based on the distribution of the iodine contrast visualized with microCT, which allowed quantification of the in vivo fluorescence of ReninSense680™.

### 4.6. Tissue Collection and Ex Vivo Fluorescent Imaging of Excised Kidneys

Mice were euthanized after in vivo microCT–FMT imaging by isoflurane overdose. Blood samples were harvested by cardiac puncture, transferred to EDTA coagulation vials and centrifuged at 4600 rpm for 10 min to collect plasma. Next, kidneys were excised, emersion-fixated in formalin and assessed for ex vivo tissue epifluorescence using the FMT system and the Odyssey^®^ CLx imaging system (LI-COR^®^ Biosciences, Lincoln, NE, USA). Probe intensity was quantified with the Odyssey infrared imaging system application software version 3.0 (LI-COR^®^ Biosciences, Lincoln, NE, USA). In order to quantify the probe intensity per kidney, the integrated intensity was divided by the shape area, resulting in counts/mm^2^ per kidney. A separate group of *Ercc1*^*d*/−^ and WT mice was sacrificed, kidneys were excised, snap frozen in liquid nitrogen and stored at −80 °C.

### 4.7. In Vitro Fluorescent Imaging of Kidney and Plasma Renin Activity

Activation of ReninSense680™ was determined in plasma (pooled plasma from C57Bl/6J mice, GeneTex, Irvine, CA, USA) and kidney lysates. Frozen kidneys of 2 WT and 4 RenKO mice were homogenized in PBS using the mortar and pestle method. Protein concentrations were determined using a Pierce BCA Protein Assay kit (Thermo Fisher Scientific, Rockford, IL, USA). Samples were pre-incubated in the presence or absence of different concentrations of the renin inhibitor aliskiren (10^−11^–10^−4^ M) at 37 °C for 30 min. Next, tissue fluorescence was assessed by incubation of plasma or kidney lysates with ReninSense680™ (end concentration 0.2 pmol/μL) at 37 °C in a humidified incubator for 30 h. Fluorescence was measured using the Odyssey^®^ CLx imaging system (excitation settings 700 nm). For background subtraction, kidney lysates of RenKO mice together with denatured kidney and plasma lysates (by heating the sample for 10 min at 70 °C) were incubated with and without ReninSense680™.

### 4.8. Statistical Analysis

Data are expressed as the means ± SEM. Differences between groups were evaluated by Student’s *t*-test or ANOVA and corrected for multiple testing by post hoc Bonferroni analysis when needed. Here, *p* < 0.05 was considered significant. All analyses were performed using IBM SPSS Statistics version 20.0 (SPSS Inc., Chicago, IL, USA).

## Data Availability

Please refer to suggested Data Availability Statements in section “MDPI Research Data Policies” at https://www.mdpi.com/ethics (accessed on 9 November 2021).
